# Effects of Xylazole on cAMP and Monoamine Neurotransmitters in Rats: In Vitro and In Vivo

**DOI:** 10.3390/cells15121120

**Published:** 2026-06-21

**Authors:** Xingyu Hou, Tianwen Ma, Yuan Zhao, Xinyu Wang, Yue Wu, Honggang Fan, Shuai Zhang

**Affiliations:** Heilongjiang Provincial Key Laboratory of Pathogenic Mechanism for Animal Disease and Comparative Medicine, College of Veterinary Medicine, Northeast Agricultural University, Harbin 150030, China; houxingyu9358@163.com (X.H.); matianwenneau@163.com (T.M.); zhaoyuanneau@163.com (Y.Z.); wangxyneau@163.com (X.W.); wuyuedyxy@163.com (Y.W.)

**Keywords:** Xylazole, cyclic AMP, monoamine neurotransmitters, microdialysis

## Abstract

**Highlights:**

**What are the main findings?**

- In P7 cortical neurons, Xylazole α_2_-adrenoceptor-dependently increases cAMP and transiently elevates extracellular dopamine, while consistently enhancing serotonin and 5-HIAA.
- In the adult rat brain, locally applied Xylazole produces region-specific changes in cAMP and monoamines—including suppression in most regions but a delayed cerebellar cAMP elevation—though these observations, at a high concentration, cannot yet be attributed to a specific receptor.

**What is the implication of the main finding?**

- These opposing, region-dependent effects reveal the complexity of Xylazole’s neurochemical actions, though the in vivo receptor mechanisms remain to be determined.

**Abstract:**

Xylazole is a sedative and analgesic agent widely used in Chinese veterinary practice, valued for its convenient administration and effectiveness. This study aimed to clarify its mechanism of action by investigating the effects on cAMP and monoamine neurotransmitters using both in vitro and in vivo rat models. In rat cortical neurons, Xylazole increased cAMP levels in a concentration- and time-dependent manner, transiently increased extracellular DA levels, which subsequently declined, consistently reduced extracellular NE levels, and enhanced extracellular 5-HT along with its metabolite 5-HIAA. In contrast, in vivo administration in adult rats reduced cAMP, DA, and NE levels across multiple brain regions, including the cerebrum, hippocampus, and brainstem, while increasing 5-HT and 5-HIAA. Notably, in the cerebellum group, cAMP was elevated after drug washout, a pattern not observed in the other brain regions. These findings reveal a striking divergence: in P7 cortical neurons, Xylazole triggers an α_2_-adrenoceptor-dependent cAMP elevation, whereas in adult brain regions, a high concentration of locally delivered Xylazole leads to predominantly inhibitory cAMP changes, with a notable delayed increase in the cerebellum. Because of the non-physiological concentration used in reverse microdialysis, the in vivo neurochemical patterns should be regarded as exploratory regional responses, not as evidence of specific receptor-mediated mechanisms.

## 1. Introduction

Xylazole [N-(2,6-dimethylphenyl)-1,3-thiazol-2-amine hydrochloride] is widely used in Chinese veterinary clinics for sedation and analgesia in animals due to its ease of administration and potent efficacy. Previous studies have shown that Xylazole provides surgical anesthesia along with effective sedative and muscle relaxant effects [1]. As an α_2_-adrenergic receptor agonist, Xylazole is similar to Xylazine, which exerts significant anesthetic effects by modulating central neurotransmitters and signal transduction systems [2,3]. Despite increasing attention on Xylazole, the mechanisms underlying its actions remain poorly understood.

The cyclic adenosine monophosphate (cAMP) signaling pathway plays a crucial role in learning and memory [4]. The temporal regulation of cAMP signaling depends mainly on adenylyl cyclase (AC) and phosphodiesterase (PDE) activities. Importantly, cAMP has several modes of action, including transmembrane signaling and signal amplification [5]. Cyclic nucleotides (cAMP, cGMP) have important functions in neurons, making these cyclic nucleotides possible mediators of anesthetic action [6]. cAMP acts mainly through the stimulation of cAMP-dependent protein kinase A (PKA), which phosphorylates several molecules, including the cAMP response element-binding protein (CREB) [7]. Ketamine has been reported to affect learning and memory by mediating the cAMP pathway and extracellular signal-regulated protein kinases (ERK) [8]. Nevertheless, in related studies, the direct role of the cAMP signaling pathway in the mechanisms of general anesthesia remains unclear. Considering the importance of cAMP, we investigated whether the cAMP signaling pathway is modulated by Xylazole and may thus represent a potential target for its anesthetic action.

The monoamine neurotransmitters, including catecholamines (dopamine, DA and norepinephrine, NE) and serotonin (5-hydroxytryptamine, 5-HT and 5-hydroxyindoleacetic acid, 5-HIAA), are a group of essential neurotransmitters [9]. In contrast to glutamate and gamma-aminobutyric acid (GABA), monoamine neurotransmitters exhibit more complex signaling mechanisms, and are integral to a wide array of physiological functions [10]. Many bodily functions also depend on the extracellular DA levels in the brain, including motor function, pituitary endocrine function, and cognitive and emotional functions. Previous scholars have found that local injection of D1 receptor (D1R) agonist or D2 receptor (D2R) agonist into olfactory tubercle (OT) enhances behavioral and cortical arousal from isoflurane anesthesia [11]. NE is an important neurotransmitter in the central nervous system (CNS) that is released by axonal terminals of NE-containing neurons and modulates neuronal activity in multiple brain regions [12]. NE can influence both macro- and microcirculation, ultimately affecting tissue oxygenation. NE administration produces a statistically significant decrease in peripheral tissue oxygenation [13].

5-HT, a biogenic monoamine, serves as an intermediate product in the metabolic pathway of tryptophan. Within the central nervous system, 5-HT plays a critical role in modulating nociception and consciousness [14]. It undergoes degradation to 5-HIAA through the monoamine oxidase (MAO) pathway, and the ratio of 5-HIAA to 5-HT is widely used as an index of 5-HT turnover in brain tissue. Substantial evidence indicates that both 5-HT and 5-HIAA contribute to the clinical effects of certain anesthetic compounds. Studies have shown that 5-HT neurons in the dorsal raphe nucleus (DRN) have a regulatory function in general anesthesia [14]. Yin et al. established that ketamine elevated the 5-HT levels in the prefrontal cortex (PFC) and hippocampus in the rat brain [15]. Lin et al. found that Paeoniflorin increased the levels of 5-HIAA in the cortex and striatum of anesthetized rats [16]. Therefore, determining the concentration changes in monoamine neurotransmitters is valuable for studying the neurochemical mechanism of Xylazole.

As an α_2_-adrenergic receptor agonist, Xylazole is structurally and pharmacologically analogous to Xylazine. However, recent evidence indicates that xylazine may interact with multiple receptor systems beyond α_2_-adrenoceptors, including muscarinic, serotonergic, and opioidergic pathways [17]. Whether Xylazole exhibits a similar multi-target profile remains unknown and warrants further investigation. In the present study, we focus on the α_2_-adrenoceptor-mediated cAMP and monoamine responses, while acknowledging that additional mechanisms may contribute to its overall pharmacological effects. Currently, few studies have focused on the molecular mechanisms of Xylazole, and no research has specifically addressed its effects on cAMP and monoamine neurotransmitters. The present study provides a comprehensive investigation of Xylazole’s neurochemical effects through the following findings and contributions: (1) the first direct in vitro and in vivo comparison of cAMP responses across multiple brain regions; (2) identification of α_2_-adrenoceptor-dependent cAMP elevation in immature cortical neurons in vitro, contrasting with cAMP suppression in the adult brain in vivo; (3) identification of a region-specific delayed cAMP elevation in the cerebellum following drug washout, a pattern distinct from that observed in the cerebrum, hippocampus, and brainstem; (4) comprehensive characterization of region-specific monoamine neurotransmitter modulation in response to Xylazole. Consequently, the present study had two distinct aims: (1) to determine whether Xylazole modulates cAMP and monoamines in an α_2_-adrenoceptor-dependent manner in P7 cortical neurons, and (2) to document, in an exploratory fashion, the regional neurochemical changes that occur in the adult brain during local high-concentration Xylazole administration, thereby generating hypotheses for future mechanistic studies at clinically relevant doses.

## 2. Materials and Methods

### 2.1. Animals, Ethics, and Drugs

Male and female Wistar rats with a mean body weight of 250 ± 25 g were purchased from the Experimental Animal Center of the Affiliated Hospital of Harbin Medical University (Harbin, China). The rats were housed in groups under a 12 h light/dark cycle at 23 °C. The rats had free access to a commercially available diet and tap water. For in vitro experiments, postnatal day 7 (P7) rat pups were obtained from timed-mated pregnant females that were housed individually and allowed to give birth naturally. The day of birth was designated as postnatal day 0 (P0), and pups of both sexes were used on P7. For in vivo microdialysis experiments, a total of 24 adult male rats were used (6 rats per brain region, with each brain region tested as a separate cohort). These rats were housed under the same conditions and were acclimatized for three weeks before experimentation. The animals were handled and maintained under strict ethical conditions according to international recommendations for animal welfare. This article does not contain any studies with human participants performed by any of the authors. The current study protocol was approved by the Northeast Agricultural University’s Animal Ethics Committee (SRM-11, China). Xylazole and Xylazine were synthesized and provided by the Laboratory of Veterinary Pharmacology, Northeast Agricultural University (Harbin, China). Yohimbine hydrochloride was purchased from MedChemExpress (MCE China, Shanghai, China).

### 2.2. Experiment Design

Primary cortical neurons were cultured in serum-free medium for 7 days. For each independent culture, cells were randomly assigned to one of seven groups: untreated control, Xylazole at 10, 20, 30, or 40 μg/mL; Xylazine 40 μg/mL (positive control); and yohimbine 10 μM (approximately 3.91 μg/mL) + Xylazole 30 μg/mL (α_2_-adrenoceptor blockade). Six independent cultures were performed per group (*n* = 6). Cell viability was first assessed by CCK-8 assay after 3 h of treatment on day 7. For cAMP and monoamine measurements, cells were sampled at 0, 5, 10, 15, 20, 25, 30, 45, 60, 90, and 120 min after treatment. A total of 24 adult male Wistar rats were used. After 3 weeks of acclimatization, rats were randomly allocated to four brain-region cohorts (cerebrum, hippocampus, cerebellum, brainstem; *n* = 6 per region) using a computer-generated random number sequence. Each rat was implanted with a microdialysis guide cannula in the assigned region and allowed to recover for 2 days. Brain regions were selected based on their distinct α_2_-adrenoceptor densities and their relevance to arousal, memory, motor coordination, and autonomic regulation. On the experimental day, a microdialysis probe was inserted under brief isoflurane anesthesia. After a 180 min equilibration period, three baseline fractions were collected (Control). Xylazole (1 mM in artificial cerebrospinal fluid, aCSF) was then delivered by reverse dialysis for 40 min at 1 μL/min. Dialysate fractions were collected every 20 min during drug perfusion (T1: 20–40 min) and for 60 min after switching back to drug-free aCSF (T2: 60–80 min; T3: 80–100 min).

To reduce bias, group allocation was hidden from both the experimenter and the analyst through independent coding, with codes revealed only after data analysis, and sample processing was counterbalanced across groups. Primary neurochemical endpoints were cAMP concentration (ELISA) and extracellular levels of DA, NE, 5-HT, and 5-HIAA (LC-MS/MS). In vitro cell viability was assessed to confirm that Xylazole at the concentrations tested did not cause cytotoxicity, thereby ruling out non-specific cellular damage as a confounding factor in the neurochemical measurements. All outcomes were treated as exploratory. Group sizes (*n* = 6 for in vitro independent cultures and *n* = 6 per brain region in vivo) were based on common practice in microdialysis and primary neuron pharmacology studies, where *n* = 5–8 per group is the established standard for detecting neurochemical changes in moderate-to-large effect sizes, not on a formal power calculation. Post-surgery, rats were monitored twice daily; body weight was recorded weekly. The study is exploratory. Criteria for potential exclusion were predefined. Humane endpoints included severe respiratory distress, inability to ambulate, or >20% body weight loss. Animals reaching these endpoints would be euthanized by CO_2_ inhalation followed by cervical dislocation. No expected or unexpected adverse events were observed in any of the animals during surgery, recovery, or microdialysis procedures.

### 2.3. Experiment Procedure

Primary neurons were obtained from the cerebral cortices of postnatal day 7 (P7) rats and cultured following the serum-free protocol of Kivell et al. [17]. P7 pups were euthanized by rapid decapitation without prior anesthesia to avoid pharmacological interference with subsequent neurochemical assays, in accordance with institutional animal ethics guidelines. Cortical tissues were dissected in ice-cold Hibernate™-A, cut into 2 mm^3^ pieces, and digested with 0.05% trypsin-EDTA containing 0.01 mg/mL DNase I for 15–30 min at 37 °C. After trituration with a siliconized Pasteur pipette, the suspension was centrifuged at 300× *g* for 5 min. Cells were resuspended in serum-free Neurobasal™-A supplemented with 2% B-27, 0.25 mM L-glutamine, 0.25 mM GlutaMax™ I, and antibiotics (osmolality 280 mOsm/kg). Tissue culture-treated multi-well plates were coated with poly-D-lysine, then pretreated with 10% heat-inactivated fetal bovine serum for 2 h (removed before seeding). Cells were seeded at 1 × 10^5^–1 × 10^6^ cells/cm^2^. Cultures were maintained at 37 °C in 5% CO_2_ with half-medium changes every 5–7 days. Neurons were used on day 7 in vitro and identified by MAP-2 immunofluorescence as described below.

Microdialysis experiments were conducted independently for each brain region using separate groups of rats. For each brain region (cerebrum, hippocampus, cerebellum, and brainstem), 6 adult male Wistar rats were used. Rats were anesthetized with isoflurane (3% for induction, 1–1.5% for maintenance, delivered in oxygen at 1 L/min) and fixed in a stereotaxic frame (RWD Life Science, Shenzhen, China). The skull was exposed, and a hole was drilled for the implantation of a microdialysis guide cannula (CMA 12, CMA/Microdialysis, Stockholm, Sweden) into the target brain region. Stereotaxic coordinates relative to bregma were according to the atlas as follows [18]: cerebrum (AP +1.0 mm, ML ±2.5 mm, DV −1.5 mm), hippocampus (AP −5.2 mm, ML ±5.0 mm, DV −3.8 mm), cerebellum (AP −11.3 mm, ML ±2.5 mm, DV −3.0 mm, targeting the cerebellar cortex), and brainstem (AP −9.5 mm, ML ±0.0 mm, DV −7.0 mm). Each rat was implanted with a guide cannula in only one brain region. Guide cannulae were secured with anchor screws and dental cement using aseptic technique; rats recovered for 2 days with peri-operative thermal support, soft bedding, and food access to reduce pain and distress.

On the experimental day, rats were briefly anesthetized with isoflurane (3%, 30–60 s) for insertion of a microdialysis probe (CMA 12 Elite; membrane length 2 mm, 20 kDa cut-off). The probe was perfused with aCSF (NaCl 147, KCl 2.7, CaCl_2_ 1.2, MgCl_2_ 0.85, Na_2_HPO_4_ 1.0 mmol/L, pH 7.4) at 1 μL/min. After a 180 min equilibration period, the three fractions immediately preceding Xylazole injection were designated as the baseline control period, which served as the control. Xylazole was administered locally by reverse dialysis through the microdialysis probe. Xylazole was dissolved in aCSF at a concentration of 1 mM (based on preliminary dose–response experiments) and perfused at 1 μL/min for 40 min. Microdialysate fractions were collected every 20 min during drug perfusion and for an additional 60 min after switching back to drug-free aCSF to monitor recovery. Fractions were collected during drug perfusion at 20–40 min (T1, early drug exposure). After switching back to drug-free aCSF at 40 min, fractions were collected at 60–80 min (T2, early recovery) and 80–100 min (T3, late recovery). The in vitro probe recoveries (mean ± SD, n = 24) were: cAMP 12.7 ± 2.3%, DA 15.6 ± 3.1%, NE 13.4 ± 2.8%, 5-HT 14.7 ± 2.7%, 5-HIAA 16.5 ± 3.3%. Probe calibration and data correction followed established protocols [19].

### 2.4. Identification of Neurons

The culture medium was aspirated, and 1 mL of paraformaldehyde-sucrose mix was added at 37 °C, followed by incubation for 10 min at room temperature. After aspirating, 1 mL of 0.1% Triton X-100 was added, and the slide was incubated for 10 min and subsequently washed three times with PBS. A total of 1 mL of 5% BSA was added and incubated for 1 h, after which MAP-2 primary antibody was applied and incubated overnight at 4 °C. The slide was then washed three times with PBS, incubated with the corresponding secondary antibody for 1 h, and washed again. Prolong Gold Antifade mounting medium was applied, the slide was inverted to remove excess solution, and it was kept in the dark for 1 h before microscopic observation (Nikon Eclipse Ti-S, Nikon Corporation, Tokyo, Japan).

### 2.5. Cell Viability Assay

Cell Counting Kit-8 (CCK-8) assay was performed to detect cell viability using the kit purchased from APExBIO (Houston, TX, USA). Cells were treated with Xylazole or 0.1% PBS (negative control) for a single 3 h exposure at 37 °C on day 7 of culture. CCK-8 reagent (10 μL) was added to each well. Cells were cultured in an incubator at 37 °C with 5% CO_2_ for 2.5 h. A microplate reader was used to measure the absorbance at 450 nm.

### 2.6. Determination of cAMP Levels

The concentration of cAMP was measured with a double-antibody sandwich enzyme-linked immunosorbent assay (ELISA) kit for biotin (Nanjing Bioengineering Institute, Nanjing, China). On day 7 of culture, neurons were treated with 10, 20, 30, or 40 μg/mL Xylazole. Untreated cells were used as negative controls. Xylazine at a concentration of 40 μg/mL was added as a positive control. To confirm α_2_-adrenoceptor involvement, an additional group was included in the same experimental batch: neurons were pretreated with yohimbine hydrochloride (10 μM) for 20 min at 37 °C before exposure to Xylazole (30 μg/mL). Cells were lysed at 0, 5, 10, 15, 20, 25, 30, 45, 60, 90, and 120 min after addition. The cells were collected by centrifugation at 300× *g* for 5 min at 4 °C. The cell pellet was washed once with ice-cold PBS and centrifuged again under the same conditions. After removing the supernatant, the cell pellet was mixed with 40 μL of RIPA lysis buffer and lysed on ice for 30 min with intermittent vortexing. To prevent protein degradation during lysis, the RIPA buffer was supplemented with protease and phosphatase inhibitors, and all steps were performed on ice. All samples were vortexed briefly for exactly 15 s each to minimize variability. The lysate was then centrifuged at 12,000× *g* for 15 min at 4 °C, and the supernatant was collected for analysis. The cAMP levels in brain microdialysate were measured using the same ELISA kit, with 40 μL of undiluted dialysate loaded per assay well, without further processing. Concentrations were expressed as pmol/mL and corrected for the in vitro recovery of each individual probe.

### 2.7. Detection of Monoamine Neurotransmitters via LC-MS/MS Analysis

The LC-MS/MS method for monoamine neurotransmitter quantification was based on our previously reported protocol [20]. For in vitro samples, at each designated time point (0, 5, 10, 15, 20, 25, 30, 45, 60, 90, and 120 min after treatment), the entire culture supernatant was collected from a dedicated well (each well was sampled only once to avoid repeated perturbation of the same culture). A 40-μL aliquot of the collected culture medium was mixed with 10 μL of a mixed internal standard working solution (DA-d_4_, NE-d_6_, 5-HT-d_4_, and 5-HIAA-d_5_, each at 50 ng/mL in 0.1% formic acid; Sigma-Aldrich, St. Louis, MO, USA) and diluted with 350 μL of ultrapure water. For brain microdialysate, a 30-μL aliquot of each fraction was mixed with 10 μL of the same internal standard working solution and 30 μL of 0.1% formic acid, vortexed, and centrifuged at 15,000× *g* for 10 min at 4 °C. An aliquot of 5 μL of the supernatant was injected directly into the LC-MS/MS system. Chromatographic separation was performed on an Xbridge BEH Amide XP column (2.1 mm × 100 mm, 1.7 μm; Waters, Milford, MA, USA) maintained at 30 °C. The mobile phase consisted of buffer A (0.1% formic acid in water) and buffer B (85% acetonitrile with 0.1% formic acid). The flow rate was 0.25 mL/min with gradient elution: 0–3.5 min, 0% B; 3.5–3.6 min, linear gradient from 0% to 30% B; 3.6–5.0 min, linear gradient from 30% to 0% B. Mass spectrometry was performed using electrospray ionization in positive mode (ESI+) with multiple reaction monitoring (MRM). Key parameters were as follows: capillary voltage 2.5 kV; source temperature 150 °C; desolvation temperature 450 °C; desolvation gas (N_2_) flow 650 L/h; cone gas flow 50 L/h; collision-induced dissociation gas pressure 3.3 × 10^−3^ mbar. Quantification was performed by the internal standard method using the peak area ratio of each analyte to its corresponding internal standard. Calibration standards were prepared by adding known amounts of analytes and internal standards to blank aCSF (for microdialysate) or blank culture medium (for in vitro samples) processed identically to the experimental samples. Calibration curves were constructed by plotting the analyte-to-internal standard peak area ratio against the nominal analyte concentration and were linear from 10 to 1000 ng/mL (R^2^ > 0.99). The limit of detection (LOD) was 4 ng/mL (S/N ≥ 3) and the limit of quantification (LOQ) was 20 ng/mL (S/N ≥ 10). Intra-day precision showed coefficients of variation < 12% for all analytes. Recoveries ranged from 89.0% to 97.3% at spiked concentrations of 100, 200, and 500 ng/mL (n = 3 per concentration). The mean extraction recovery and matrix effect for each internal standard were assessed at the working concentration (5 ng/mL in the final sample) and found to be within 90–110%.

### 2.8. Statistical Analysis

Data are presented as mean ± SEM and analyzed using GraphPad Prism 8.2 (GraphPad Software Inc., San Diego, CA, USA). For in vitro experiments, data were obtained from six independent cultures (*n* = 6). Two-way ANOVA (group × time) with Sidak’s post hoc test was used to compare all treatment groups to the control group, the yohimbine + Xylazole group to the Xylazole alone group, and the Xylazine group to the control group where applicable, at each time point. For in vivo microdialysis, each brain region was analyzed independently. All data were obtained from repeated sampling of the same animals (*n* = 6 per brain region) over time. One-way repeated-measures ANOVA with Sidak’s post hoc test compared levels across the four time periods (Control, T1, T2, T3) within each brain region. A *p*-value < 0.05 was considered statistically significant.

## 3. Results

### 3.1. Rat Cortical Neurons Culture and Identification

**Figure 1:**
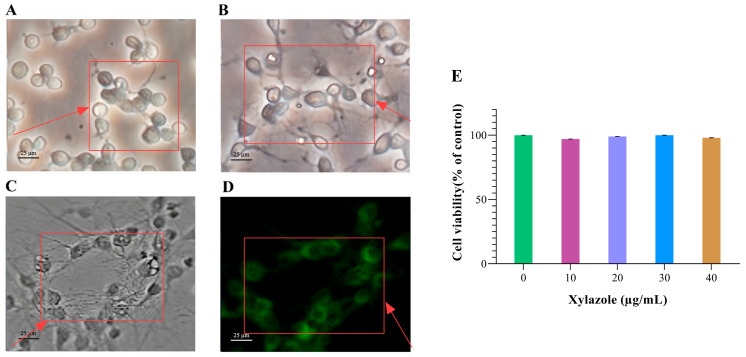
Observation of primary cortical neurons from postnatal day 7 (P7) rats in vitro. (**A**–**C**) Phase-contrast images at 3 h, 3 days, and 7 days after plating. (**D**) Immunofluorescence staining for MAP2, a neuronal marker, confirming neuronal identity on day 7 in culture. (**E**) Effects of different concentrations of Xylazole on cortical neuron viability assessed by CCK-8 assay. Data are presented as means  ±  SEM (*n* = 6).

Rat cortical neurons were observed under a phase-contrast microscope. After 3 h of incubation and culture, a few adherent cells were observed on culture plates. The cells attached to the plate as single cells or small clusters. Most of the cortical neurons appeared transparent and were uniformly distributed (Figure 1A). On the third day of culture, the cortical neurons exhibited an elongated morphology with a high refractive index, neurites began to appear and formed a network (Figure 1B). Following seven days of culture, a significantly higher number of neuronal cell bodies appeared with intact nuclei and distinct nucleoli. It was observed that neurites further extended and formed intercellular connections (Figure 1C). The cultured neurons were positive for MAP2, a neuronal marker, as shown by laser confocal microscopy (Figure 1D).

### 3.2. Effects of Different Xylazole Concentrations on Rat Cortical Neuronal Viability

The CCK-8 assay was used to test the effects of Xylazole on rat cortical neuron viability. No significant differences were observed in the Xylazole group (10, 20, 30, and 40 μg/mL) compared with the control group (0.1% PBS) (Figure 1E). The results suggested that Xylazole had no significant effects on cortical neuron viability. Cell viability was assessed at day 7 to match the time point used for all subsequent cAMP and monoamine measurements, thereby confirming that the concentrations employed in the neurochemical assays did not cause cytotoxicity at this developmental stage.

### 3.3. Effects of Different Xylazole Concentrations on cAMP in Rat Cortical Neurons

**Figure 2:**
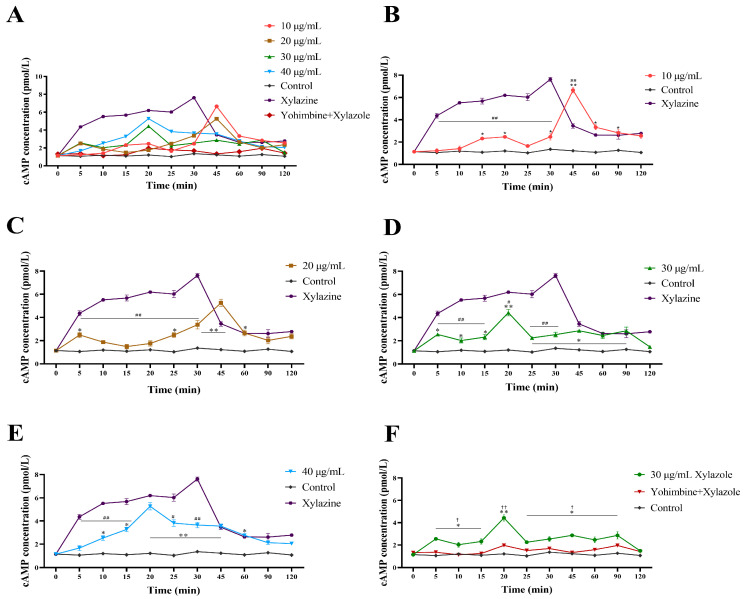
Effects of Xylazole on the cAMP concentration in vitro. (**A**–**E**) Temporal changes in cAMP levels following treatment with Xylazole (10, 20, 30, 40 μg/mL) compared with untreated control and Xylazine (40 μg/mL, positive control). (**F**) Effects of pretreatment with the α_2_-adrenoceptor antagonist yohimbine (10 μM, 20 min prior) on Xylazole (30 μg/mL)-induced cAMP elevation. Data are presented as means  ±  SEM (*n* = 6). * *p* < 0.05, ** *p* < 0.01 vs. control group. ^#^ *p* < 0.05, ^##^ *p* < 0.01 vs. Xylazine group. ^†^ *p* < 0.05, ^††^ *p* < 0.01 vs. Yohimbine + Xylazole group.

To study the effects of Xylazole on cAMP production, we measured cAMP concentration and observed that Xylazole treatment led to a concentration- and time-dependent increase in cAMP levels. The temporal changes in cAMP levels following treatment with different concentrations of Xylazole are shown in Figure 2. The cAMP concentration in cortical neurons treated with 10 μg/mL Xylazole increased after 10 min (Figure 2B). The cAMP levels in cortical neurons treated with 20 μg/mL and 30 μg/mL Xylazole increased significantly after 5 min (Figure 2C,D) (*p* < 0.05). Additionally, the cAMP concentration was significantly higher in the 30 μg/mL and 40 μg/mL Xylazole groups at 20 min compared with control (*p* < 0.01) (Figure 2D). The 10 μg/mL and 20 μg/mL groups reached peak concentrations at 45 min (*p* < 0.01) (Figure 2A,C), while the 30 μg/mL and 40 μg/mL groups reached peak concentrations at 20 min (*p* < 0.01). The change in cAMP concentration in all groups leveled off after 60 min. Pretreatment with the α_2_-adrenoceptor antagonist yohimbine (10 μM) completely abolished the Xylazole-induced elevation of cAMP. In the yohimbine + Xylazole group, during 5–90 min, cAMP levels in 30 μg/mL Xylazole group were significantly higher than the yohimbine + Xylazole group (Figure 2F) (*p* < 0.05), cAMP levels at all time points remained comparable to those of the control group (*p* > 0.05). The temporal pattern of cAMP change in each Xylazole-treated group was similar to that of the Xylazine-treated group.

### 3.4. Effects of Xylazole on cAMP in Different Brain Regions of Rats

**Figure 3:**
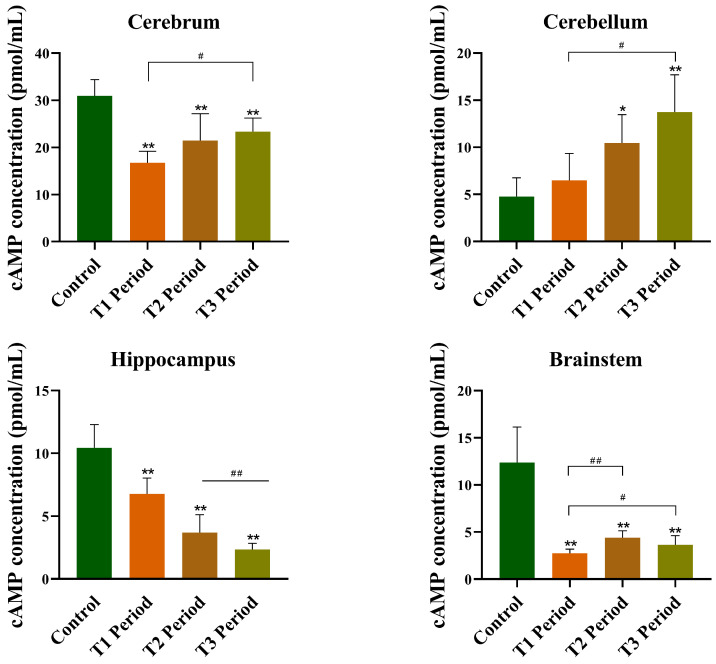
Effects of Xylazole on cAMP concentration in different brain regions of rats in vivo. T1 (20–40 min, during Xylazole perfusion), T2 (60–80 min, early recovery), T3 (80–100 min, late recovery). Data are presented as means  ±  SEM (*n* = 6). * *p* < 0.05, ** *p* < 0.01 vs. control group. ^#^ *p* < 0.05, ^##^ *p* < 0.01 vs. T1 period. Detailed statistics for each brain region: Cerebrum—T1: −45.95%, T2: −30.70%, T3: −24.49% vs. Control (all *p* < 0.01). Hippocampus—T1: −35.20%, T2: −64.64%, T3: −80.92% vs. Control (all *p* < 0.01). Brainstem—significant inhibition at all time points (*p* < 0.01). Cerebellum—T1: +35.66% (*p* > 0.05), T2: +118.81% (*p* < 0.05), T3: +187.13% (*p* < 0.01).

Xylazole exerted region-specific effects on cAMP levels (Figure 3). T1 corresponds to the 20–40 min drug perfusion period, T2 to 60–80 min (early recovery), and T3 to 80–100 min (late recovery). In the cerebrum, hippocampus, and brainstem, Xylazole significantly inhibited cAMP production across all time periods (*p* < 0.01 vs. Control). In the cerebrum, cAMP levels decreased by 45.95% at T1 and partially recovered to 24.49% below baseline by T3. In the hippocampus, the reduction was progressive, declining from 35.20% at T1 to 80.92% at T3. The brainstem showed a similar inhibitory pattern. In contrast, the cerebellum exhibited a delayed and progressive cAMP elevation. The increase was not statistically significant at T1 (35.66%, *p* > 0.05) but became significant at T2 (118.81%, *p* < 0.05) and highly significant at T3 (187.13%, *p* < 0.01).

### 3.5. Effects of Different Xylazole Concentrations on Monoamine Neurotransmitters in Rat Cortical Neurons

**Figure 4:**
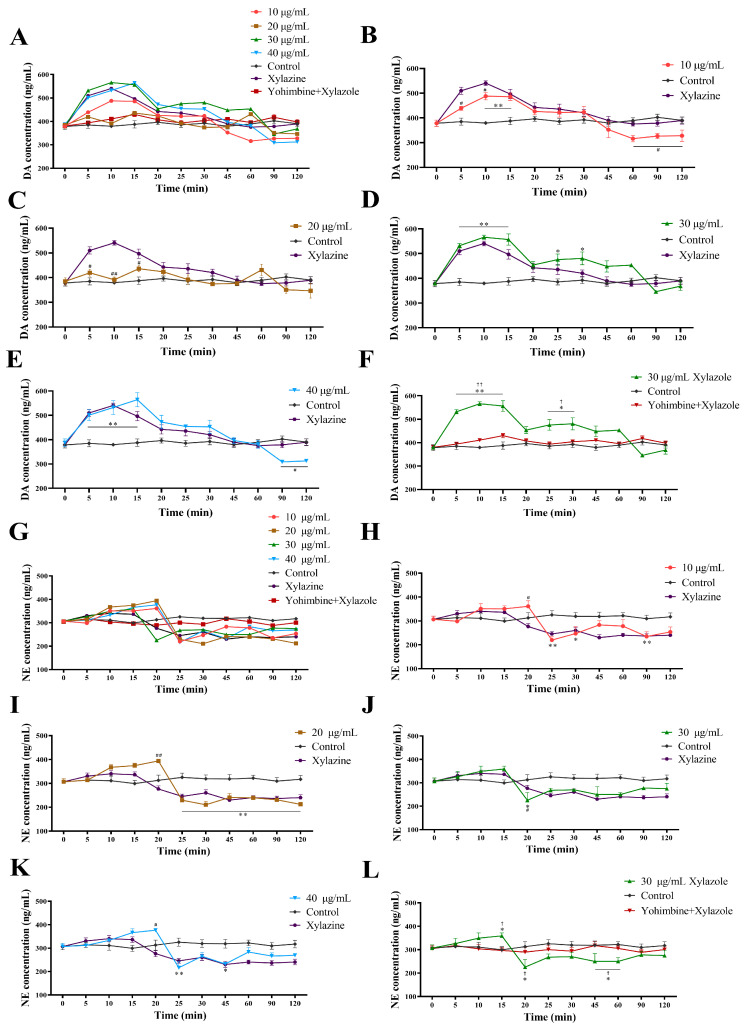
Effects of Xylazole on the DA and NE concentrations in vitro. (**A**–**F**) The DA concentration; (**G**–**L**) the NE concentration. Data are presented as means  ±  SEM (*n* = 6). * *p* < 0.05, ** *p* < 0.01 vs. control group. ^#^ *p* < 0.05, ^##^ *p* < 0.01 vs. Xylazine group. ^†^ *p* < 0.05, ^††^ *p* < 0.01 vs. Yohimbine + Xylazole group.

**Figure 5:**
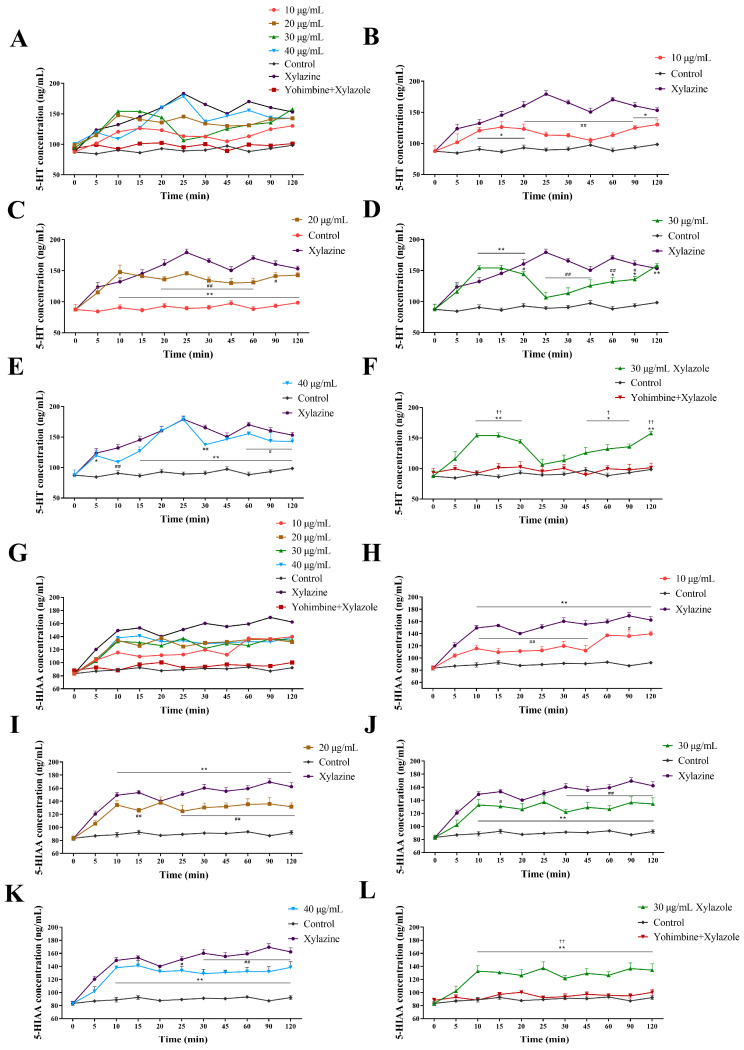
Effects of Xylazole on the 5-HT and 5-HIAA concentrations in vitro. (**A**–**F**) The 5-HT concentration; (**G**–**L**) the 5-HIAA concentration. Data are presented as means  ±  SEM (*n* = 6). * *p* < 0.05, ** *p* < 0.01 vs. control group. ^#^ *p* < 0.05, ^##^ *p* < 0.01 vs. Xylazine group. ^†^ *p* < 0.05, ^††^ *p* < 0.01 vs. Yohimbine + Xylazole group.

Xylazole produced a biphasic extracellular DA response in cortical neurons, characterized by a rapid transient increase followed by a sustained decline (Figure 4A–E). Significant increases over control were observed at 5–15 min in the 30 and 40 μg/mL groups and at 10–15 min in the 10 μg/mL group (*p* < 0.01); the 20 μg/mL group showed a non-significant increase. All groups peaked at 10–15 min, after which DA concentrations declined and did not differ significantly from control beyond 20 min. In all Xylazole-treated groups, extracellular NE levels remained stable during the first 15–20 min before decreasing significantly (*p* < 0.01) and reaching a plateau after 30 min (Figure 4G–K). Xylazole treatment produced a sustained increase in extracellular 5-HT levels across all concentration groups (Figure 5A–E). No significant changes were observed during the first 5 min. Significant elevations emerged at 10–15 min (*p* < 0.01), peaked, and then declined slightly, but remained significantly elevated after 45 min compared to the control group. 5-HIAA concentrations increased significantly starting at 10 min in all Xylazole-treated groups (*p* < 0.01) and remained elevated throughout the 120 min observation period (Figure 5G–K). The overall changes in monoamine levels in the Xylazole-treated groups were similar to those observed in the Xylazine-treated group. Yohimbine pretreatment abolished the Xylazole-induced changes in monoamine neurotransmitters. In the yohimbine + Xylazole group, monoamine neurotransmitter levels showed no transient increase and remained comparable to the control group throughout the observation period (Figure 4F,L and Figure 5F,L) (*p* > 0.05).

### 3.6. Effects of Xylazole on Monoamine Neurotransmitters in Different Brain Regions of Rats

**Figure 6:**
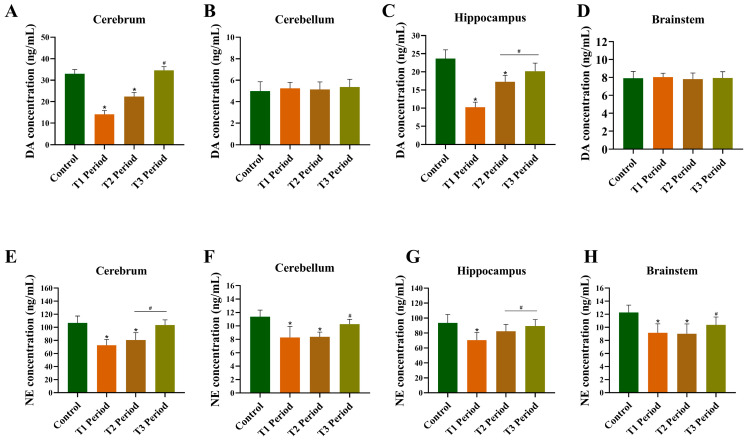
Effects of Xylazole on the DA and NE concentrations in vivo. (**A**–**D**) The DA concentration; (**E**–**H**) the NE concentration. Data are presented as means  ±  SEM (*n* = 6). * *p* < 0.05, ** *p* < 0.01 vs. control group. ^#^ *p* < 0.05, ^##^ *p* < 0.01 vs. T1 Period. DA: Cerebrum T1 −57.29% (*p* < 0.05), Hippocampus T1 −56.64% (*p* < 0.05); Cerebellum and Brainstem: no significant change. NE: Cerebrum −31.95%, Cerebellum −27.25%, Hippocampus −24.70%, Brainstem −25.34% (all *p* < 0.05 at T1).

Figure 6 shows the DA and NE concentrations across brain regions. Xylazole administration produced region-specific decreases in extracellular DA and NE levels (Figure 6). DA concentrations decreased significantly in the cerebrum (57.29%) and hippocampus (56.64%) at T1 (*p* < 0.05), partially recovered at T2, and returned to baseline by T3. No significant DA changes were observed in the cerebellum or brainstem. In contrast, NE levels decreased significantly across all four brain regions at T1 (24.70–31.95%, *p* < 0.05) and gradually recovered during T2–T3.

**Figure 7:**
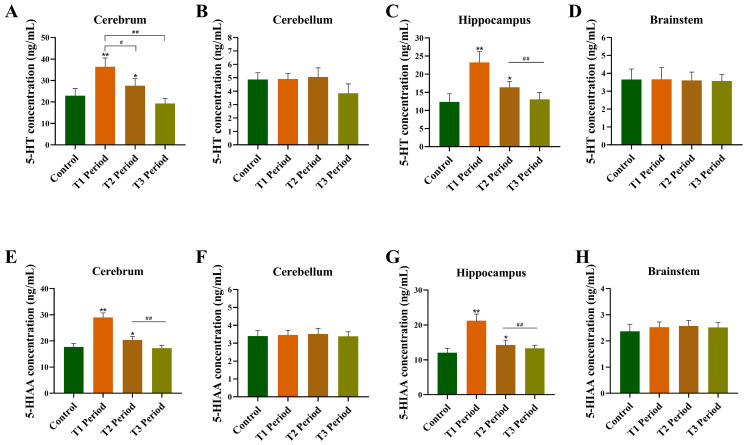
Effects of Xylazole on the 5-HT and 5-HIAA concentrations in vivo. (**A**–**D**) The 5-HT concentration; (**E**–**H**) the 5-HIAA concentration. Data are presented as means  ±  SEM (*n* = 6). * *p* < 0.05, ** *p* < 0.01 vs. control group. ^#^ *p* < 0.05, ^##^ *p* < 0.01 vs. T1 Period. 5-HT: Cerebrum T1 +58.93% (*p* < 0.01), Hippocampus T1 +88.13% (*p* < 0.01); Cerebellum and Brainstem: no significant change. 5-HIAA: Cerebrum T1 +64.16% (*p* < 0.05), Hippocampus T1 +76.15% (*p* < 0.05); Cerebellum and Brainstem: no significant change.

Both 5-HT and 5-HIAA levels increased significantly in the cerebrum and hippocampus during T1 (5-HT: 58.93% and 88.13%, *p* < 0.01; 5-HIAA: 64.16% and 76.15%, *p* < 0.05), and returned to baseline by T3 (Figure 7). No significant changes in either analyte were observed in the cerebellum or brainstem across any time period. The parallel elevation of 5-HT and 5-HIAA in the cerebrum and hippocampus suggests region-specific enhancement of serotonergic indices during Xylazole perfusion.

## 4. Discussion

The modulation of cAMP signaling by various anesthetics is well established. Previous studies have shown that lidocaine increases cAMP levels in the brain of rats [21], researchers have reported similar findings with sevoflurane anesthesia, which increases cAMP accumulation more than in the model group [22]. Furthermore, propofol can reduce the level of cAMP in the hippocampus of rats [23]. However, further research is needed to determine the specific mechanisms and the relationship between Xylazole and the cAMP signaling pathway. In this study, we observed contrasting cAMP responses depending on the experimental model: Xylazole elevated cAMP in rat cortical neurons but decreased it in most adult brain regions in vivo. A notable exception was the cerebellum, where cAMP increased during the later sampling periods (T2–T3). This apparent discrepancy highlights the complexity of drug action in intact neural circuits. However, the high local concentration of Xylazole used in the microdialysis experiments precludes any definitive attribution of these in vivo effects to specific receptor mechanisms.

In contrast to the in vivo findings, the in vitro experiments provided clear evidence for α_2_-adrenoceptor involvement. The present in vitro antagonist experiments demonstrated that Xylazole-induced cAMP elevation in P7 cortical neurons was completely blocked by the selective α_2_-adrenoceptor antagonist yohimbine, indicating that this effect is α_2_-adrenoceptor-dependent in this in vitro model. This finding is consistent with previous reports that α_2_-adrenoceptors can couple to cAMP elevation rather than inhibition in certain cell types, particularly in neuron-like cells such as PC12 cells [24]. In such systems, α_2_-adrenoceptor-mediated cAMP elevation has been attributed to Gβγ subunit activation of adenylyl cyclase isoforms II and IV, which are highly expressed in the brain, or to alternative pathways involving arachidonic acid metabolism and protein kinase A activation [25,26]. These non-canonical signaling mechanisms, previously characterized in reduced cellular systems, provide a reasonable mechanism for the α_2_-adrenoceptor-dependent cAMP elevation observed in our in vitro experiments. While our in vitro data demonstrate that α_2_-adrenoceptor activation can elevate cAMP in P7 neurons, the cellular mechanism underlying the delayed cerebellar cAMP rise in vivo cannot be determined without in vivo receptor blockade—it could involve α_2_-adrenoceptors, other targets engaged at the high Xylazole concentration, or indirect circuit effects.

The pronounced and delayed elevation of cAMP in the cerebellum, which contrasts with the suppression observed in the cerebrum, hippocampus, and brainstem, is a notable observation of the present study. However, the high drug concentration precludes definitive attribution of this effect to any specific receptor mechanism. Although no specific mechanism can be confirmed, we note that the NE levels in the cerebellum did not exhibit a rebound increase during the T2–T3 periods (Figure 6F). This observation argues against a β-adrenoceptor-mediated rebound mechanism, but does not, on its own, identify the pathway responsible. The exact molecular basis for this region-specific cAMP response remains to be elucidated. Future studies employing region-specific pharmacological blockade, as well as primary cerebellar granule cell cultures, would help determine the receptor and circuit basis of this phenomenon.

A fundamental limitation of the in vivo reverse microdialysis experiments must be acknowledged. The Xylazole concentration in the perfusate (1 mM) is non-physiological and far exceeds brain concentrations achievable during systemic veterinary administration. At this concentration, the drug inevitably diffuses beyond the target region and acts at multiple receptor systems, making it impossible to attribute the observed neurochemical changes specifically to α_2_-adrenoceptor activation. Therefore, the in vivo findings should be interpreted strictly as descriptive observations of regional neurochemical responses to local Xylazole administration, without inference regarding specific receptor mechanisms. This limitation also highlights a broader need in the field: pharmacokinetic data on Xylazole’s brain penetration would be essential to guide the selection of clinically relevant concentrations for future in vivo mechanistic studies.

The use of P0–P7 rodent cortical neurons for in vitro pharmacological studies is a well-established approach in neuropharmacology, including investigations of α_2_-adrenoceptor signaling and general anesthetic mechanisms [17,27,28]. At this developmental stage, cortical neurons have completed neurogenesis and migration, express functional α_2_-adrenoceptors, and are capable of forming synaptic networks in culture, while retaining sufficient viability for serum-free culture without astrocyte feeder layers. The present yohimbine antagonist experiments confirmed that Xylazole-induced cAMP elevation in these P7 neurons is mediated through α_2_-adrenoceptor activation, validating the utility of this model for studying Xylazole’s receptor-dependent mechanisms. However, the differences between in vitro and in vivo cAMP responses highlight the limitations of using isolated neurons to model the intact brain. The use of P7 cortical neurons versus adult brain tissue introduces differences in both developmental stage and network context that may contribute to the divergent cAMP responses. First, cultured neurons lack tonic inputs from upstream nuclei, such as locus coeruleus, that strongly influence drug responsiveness [29]. In the intact brain, the net effect of an α_2_-adrenergic agonist emerges from the dynamic interplay between local receptor activation, presynaptic modulation, and circuit-level feedback loops—all of which are absent in dissociated cultures [29,30]. This absence of network integration is likely a dominant contributing factor accounting for the opposing cAMP responses observed between the two experimental settings. Second, even at the cellular level, rat cortical neurons exhibit distinct α_2_ receptor coupling compared to adult neurons, with immature cells favoring Gβγ-mediated adenylyl cyclase activation that elevates cAMP [31]. While developmental differences in α_2_-adrenoceptor coupling may contribute to the in vitro cAMP elevation, the absence of network-level modulation in dissociated cultures likely accounts for the divergent cAMP responses between the two experimental settings. Definitive resolution of the relative contributions of these factors will require further investigation. It should also be noted that P7 cortical cultures inevitably contain glial cells, which may influence neuronal cAMP and monoamine responses through gliotransmitter release, metabolic coupling, or direct α_2_-adrenoceptor signaling. Whether glial components contribute to the observed α_2_-adrenoceptor-dependent cAMP elevation remains to be determined. Future studies using purified neuronal cultures or neuron-glia co-cultures would help clarify this issue. Additionally, extending cortical neuron cultures to 2–3 weeks in vitro would help determine whether the cAMP-elevating response reflects an immature neuronal phenotype or is a stable property of these cells. If mature cultured neurons retain this response, a developmental mechanism would be favored; if the response diminishes, the absence of intact network inputs becomes a more likely explanation. It should also be noted that the in vitro concentrations represent cumulative release over the sampling interval in a static culture system lacking the continuous clearance mechanisms present in vivo. Therefore, absolute in vitro concentrations should not be directly compared with in vivo microdialysate values.

DA serves as the direct biosynthetic precursor of norepinephrine [32], therefore, changes in DA availability can influence NE levels. Accordingly, the regulation of DA release (either reducing or increasing) has a significant impact on NE concentration. Our results show that Xylazole influenced the extracellular concentrations of DA and NE in rat cortical neurons. For DA, levels increased from 5 to 15 min, after which they gradually declined in all four groups. Once they reached 90 min, the levels tended to remain stable. In comparison with the control group, the DA concentration during the 10–15 min intervals was significantly different. NE showed a similar pattern, with an initial increase followed by a decrease. Groups treated with 30 μg/mL Xylazole reached the lowest level after 20 min, while the other groups took 25–30 min. After 30 min, all groups leveled off and showed highly significant differences. In vivo, during local Xylazole administration, NE levels in the cerebrum, cerebellum, brainstem, and hippocampus exhibited a trend of first decreasing and then increasing. The lowest NE concentration in most brain regions occurred during the T1 period, and the minimum NE concentration in each brain region was significantly decreased compared to the control group.

Many studies suggest that DA receptors play a role in anesthesia. Previous findings indicate that DA influences reward processing and cognitive functions via circuits like the ventral tegmental area-PFC and PFC-nucleus accumbens. Dysfunctional receptors alter the activity of PFC pyramidal neurons [33]. Our results show that Xylazole gradually reduced extracellular DA over 120 min. The observed decline in DA and NE levels in specific brain regions is a consistent regional pattern during local high-concentration Xylazole perfusion. Whether this reflects presynaptic α_2_-adrenoceptor activation, off-target effects, or local feedback inhibition cannot be determined from the present data. However, direct evidence for this pathway is lacking, and alternative explanations cannot be ruled out. The transient DA increase followed by sustained decline is consistent with α_2_-adrenoceptor-mediated modulation of DA synthesis and its subsequent conversion to NE. The complete blockade of Xylazole-induced DA, NE, 5-HT, and 5-HIAA changes by yohimbine indicates that these monoaminergic effects are all α_2_-adrenoceptor-dependent in vitro. In the experiment, the changes in DA concentration in the cerebrum and hippocampus were generally similar to those of NE, whereas the changes in DA concentration in the cerebellum and brainstem were not significant. Similar trends in DA and NE levels during local Xylazole administration may be attributed to DA serving as a precursor for NE synthesis.

Previous studies have demonstrated that α_2_-adrenergic receptor agonists reduce NE levels. This is because α_2_-agonists activate presynaptic Na^+^-K^+^-ATPase, which reduces Ca^2+^ influx and lowers intracellular Ca^2+^. Additionally, the reduction of Ca^2+^ influences the fusion of vesicles with the presynaptic membrane, leading to reduced NE release [34,35]. However, MAO and catechol-O-methyltransferase (COMT) continue to metabolize intracellular NE, further reducing NE stores. This finding is consistent with the results of Changmin et al. [1], which revealed that Xylazole inhibited NE in venous blood. Our in vitro data show that Xylazole reduces NE release, consistent with the known effects of α_2_ agonists. Unlike DA and NE, 5-HT and 5-HIAA increased in both models and in the cerebrum and hippocampus (Figure 5 and Figure 7), a pattern consistent with reports that other anesthetics such as ketamine also increase serotonin release [36,37]. 5-HT and 5-HIAA increased significantly in the cerebrum and hippocampus but did not change in the cerebellum or brainstem.

A methodological consideration that warrants particular attention is the extracellular 5-HIAA/5-HT ratio observed in the present microdialysis experiments. We fully acknowledge that, even though the baseline (Control) samples were collected after a standard 180 min equilibration period, the 5-HIAA/5-HT ratio in our study differs substantially from the classical steady-state values reported in the literature, possibly reflecting residual local tissue perturbation caused by probe insertion and the brief isoflurane anesthesia used during the procedure [38,39]. During Xylazole perfusion (T1), the Xylazole-induced surge of 5-HT release may temporarily outpace its metabolic conversion to 5-HIAA via MAO-A, resulting in a transient narrowing of the 5-HIAA/5-HT ratio. The return of both analytes toward baseline during the T2–T3 recovery periods is consistent with this kinetic interpretation [40,41]. Additionally, the metabolic conversion of 5-HT to 5-HIAA is primarily catalyzed by MAO-A. Given that Xylazole induces a rapid and substantial increase in 5-HT release, it is plausible that the capacity of MAO-A to immediately metabolize this surge of 5-HT is transiently exceeded, contributing to the observed narrowing of the 5-HIAA/5-HT ratio. This hypothesis warrants dedicated investigation in future experiments. It should also be noted that DA levels exceeded 5-HT levels in certain brain regions under our experimental conditions, a pattern that differs from classical steady-state observations. This may reflect a combination of the specific stereotaxic coordinates sampled, strain differences (Wistar rats), the acute pharmacological effects of Xylazole on dopaminergic systems, and local tissue perturbation from probe insertion. While we have discussed several potential contributing factors above, we cannot fully exclude the possibility that unrecognized methodological or biological factors may have influenced these measurements. We, therefore, recommend caution in interpreting absolute baseline values. Future studies using zero-net-flux quantitative microdialysis would help establish whether the observed ratios reflect genuine biological phenomena under our experimental conditions.

Several limitations should be acknowledged. First, as discussed in detail above, the non-physiological concentration of Xylazole (1 mM) in the perfusate fundamentally limits the mechanistic interpretation of all in vivo findings. The critical assessment of the concentration issue during peer review prompted us to fundamentally reconsider the scope of our conclusions, and we are grateful for this guidance. Second, no α_2_-adrenoceptor antagonist was used in the in vivo microdialysis experiments. Therefore, the mediation of the observed in vivo neurochemical effects via α_2_ receptors, while pharmacologically plausible given the structural and pharmacological similarity of Xylazole to the known α_2_-agonist xylazine, remains to be directly validated in the intact brain. Future studies employing local co-perfusion of yohimbine or other selective α_2_-adrenoceptor antagonists are essential to establish causal receptor mediation. Third, the relative contributions of developmental stage and network context cannot be disentangled in the present design; future studies using adult neuronal cultures or brain slices are needed to isolate these variables. Fourth, the in vitro antagonist experiments did not include a yohimbine-alone control group. While the complete normalization of all endpoints to control levels in the yohimbine + Xylazole group provides internal evidence against independent yohimbine activity, a dedicated antagonist-alone group would further strengthen the conclusions. A yohimbine-alone control group will be included in our future studies to formally exclude any potential interference from independent pharmacological activity of yohimbine. Fifth, group sizes were based on field-standard conventions rather than formal a priori power analysis. Consequently, the study may have been underpowered to detect smaller effect sizes. The observed effect sizes for cAMP and monoamine changes were generally large and achieved statistical significance, providing confidence in the main findings. However, more subtle neurochemical changes may have been missed, and future confirmatory studies should employ a priori power calculations based on the effect sizes reported here to determine optimal sample sizes.

## 5. Conclusions

In conclusion, this study provides the first direct evidence that Xylazole can elevate cAMP in an α_2_-adrenoceptor-dependent manner in P7 rat cortical neurons, accompanied by a transient increase in extracellular dopamine and sustained serotonin enhancement. When locally delivered at a high concentration to the adult brain, Xylazole induces region-specific alterations in cAMP and monoamine levels, including a pronounced delayed cAMP increase unique to the cerebellum. Critically, these in vivo observations cannot be attributed to α_2_-adrenoceptor activation because of the supraphysiological concentration and the absence of in vivo receptor blockade. The in vitro findings thus establish a mechanistic framework, while the in vivo data represent hypothesis-generating regional profiles that require validation under clinically relevant conditions with direct pharmacological antagonism.

## Data Availability

The data presented in this study are available on request from the authors (X.H., H.F. and S.Z.).

## Abbreviations

The following abbreviations are used in this manuscript:

5-HIAA	5-hydroxyindoleacetic acid
5-HT	5-hydroxytryptamine
AC	adenylyl cyclase
ANOVA	analysis of variance
cAMP	cyclic adenosine monophosphate
cGMP	cyclic guanosine monophosphate
CNS	central nervous system
CREB	cAMP response element-binding protein
D1R	D1 receptor
D2R	D2 receptor
DA	dopamine
ERK	extracellular signal-regulated protein kinases
GABA	gamma-aminobutyric acid
LOD	limit of detection
LOQ	limit of quantification
MAO	monoamine oxidase
NE	norepinephrine
OT	olfactory tubercle
PDE	phosphodiesterase
PFC	prefrontal cortex
PKA	Protein kinase A
Xylazole	N-(2,6-dimethylphenyl)-1,3-thiazol-2-amine hydrochloride
